# Seasonal Cycles in a Seaweed Holobiont: A Multiyear Time Series Reveals Repetitive Microbial Shifts and Core Taxa

**DOI:** 10.1111/1462-2920.70062

**Published:** 2025-02-27

**Authors:** Chantal Marie Mudlaff, Florian Weinberger, Luisa Düsedau, Marjan Ghotbi, Sven Künzel, Guido Bonthond

**Affiliations:** ^1^ Department of Marine Ecology GEOMAR Helmholtz Centre for Ocean Research Kiel Kiel Germany; ^2^ Faculty of Mathematics and Natural Sciences Christian‐Albrechts‐Universität zu Kiel Kiel Germany; ^3^ Section Benthic Ecology Alfred Wegener Institute Helmholtz Centre for Polar and Marine Research Bremerhaven Germany; ^4^ Department of Evolutionary Genetics Max Planck Institute for Evolutionary Biology Plön Germany; ^5^ Institute for Chemistry and Biology of the Marine Environment (ICBM), School of Mathematics and Science Carl von Ossietzky Universität Oldenburg Oldenburg Germany

**Keywords:** epibiota, holobiont, host–microbe, macroalga, seasonality, seaweed

## Abstract

Seasonality is an important natural feature that drives cyclic environmental changes. Seaweed holobionts, inhabiting shallow waters such as rocky shores and mud flats, are subject to seasonal changes in particular, but little is known about the influence of seasonality on their microbial communities. In this study, we conducted a three‐year time series, sampling at two‐month intervals, to assess the seasonality of microbial epibiota in the seaweed holobiont *Gracilaria vermiculophylla*. Our results reveal pronounced seasonal shifts that are both taxonomic and functional, oscillating between late winter and early summer across consecutive years. While epibiota varied taxonomically between populations, they were functionally similar, indicating that seasonal variability drives functional changes, while spatial variability is more redundant. We also identified seasonal core microbiota that consistently (re)associated with the host at specific times, alongside a permanent core that is present year‐round, independent of season or geography. These findings highlight the dynamic yet resilient nature of seaweed holobionts and demonstrate that their epibiota undergo predictable changes. Therewith, this research offers important insights into the temporal dynamics of seaweed‐associated microbiota and demonstrates that the relationship between seaweed host and its epibiota is not static but naturally subject to an ongoing seasonal succession process.

## Graphical abstract

The red seaweed *Gracilaria vermiculophylla* holobiont exhibits strong seasonal succession. Its epibiotic microbial community shifts annually from winter to summer and back. The holobiont features a permanent core of year‐round present taxa and distinct seasonal cores, with taxa that consistently return during winter or summer.

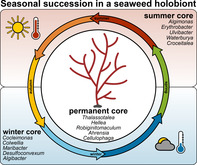

## 1 Introduction

Seasonality is a global environmental feature which plays an important role in structuring communities in terrestrial and aquatic environments (White and Hastings 2020). To communities, seasonality is a natural source of variability, characterised by cyclic changes in temperature and photoperiod, but also by other variables such as salinity, rainfall, phytoplankton blooms, anthropogenic stressors, upwelling and nutrient pulses (Lisovski et al. 2017). Seasonal fluctuations are also prevalent in free‐living microbial communities (Fuhrman et al. 2015; Gilbert et al. 2012) and microbial communities associated with various hosts (Sharp et al. 2017; Ferguson et al. 2018; Gobbi et al. 2020; Risely et al. 2021), including seaweeds (Bengtsson et al. 2010; Park et al. 2022; Tujula et al. 2010).

Seaweeds are typically found in coastal habitats, such as rocky shores and intertidal mudflats, which experience strong seasonal forcing (Benincà et al. 2015). Naturally, seaweed holobionts are surrounded by microbial life. The algal surface is in direct contact with the surrounding water and acts as a substrate on which microorganisms can settle (Wahl et al. 2012). These colonising communities are typically dominated by bacteria but also include microalgae, fungi, protists and viruses (Egan et al. 2013; Van Der Loos et al. 2019). On the interface between the seawater and the inner tissue of the seaweed, epibiota form a dynamic biofilm, which has also been termed the ‘second skin’, and influences the host physiologically, chemically and biologically (Wahl et al. 2012). These epibiota include (opportunistic) pathogens but also beneficial microbes that promote the host's development and fitness, such as, for example, sporulation (Weinberger et al. 2007) or morphogenesis (Weiss et al. 2017, reviewed in Egan et al. 2013), pathogen recognition and chemically mediated defence mechanisms (Li et al. 2022; Longford et al. 2019; Rao et al. 2007; Saha and Weinberger 2019).

Epiphytic communities are complex, and their structure depends on host morphology (Lemay et al. 2021), varies by species (Lachnit et al. 2009, 2011) and even lifecycle stage (Lemay et al. 2018; Bonthond et al. 2022), and differs along the algal thallus (Serebryakova et al. 2018; Paix et al. 2020), illustrating the specific and diverse niches provided by the host and the microbial partners. The holobiont is exposed to variable environmental conditions, which may alter the epibiota composition either directly, or indirectly via host physiological responses to the changing environment. For instance, seaweed epibiota have been found to strongly vary with salinity (Stratil et al. 2014; Van Der Loos et al. 2023) and temperature (Stratil et al. 2013; Bonthond et al. 2023). Thus, seaweed epibiota are shaped by a combination of host and environment. As both environmental conditions and host physiology are seasonal, it is not surprising that seasonal patterns have been detected in seaweed epibiota (Bengtsson et al. 2010; Park et al. 2022; Tujula et al. 2010). While it is informative to document microbial changes within the holobiont from one season to another, it is important to distinguish which changes are repetitive. Such cyclic patterns reflect an ongoing interaction between host and microbiota, with long multigenerational histories. Microbial taxa that are present irrespective of season, or that return interannually, may represent important core symbionts (beneficial or harmful). Studying holobionts during different seasons across several years may resolve such permanent and/or seasonal core microbiota and therewith contribute to the identification of important host–microbe associations and to better understand the complex dynamics within the holobiont.

*Gracilaria vermiculophylla* is a well‐studied holobiont. This perennial rhodophyte is native to the North‐West Pacific (Kim et al. 2010; Krueger‐Hadfield et al. 2017, 2021) but has become invasive across the Northern Hemisphere, including the North American Eastern Pacific coast southward to Mexico (Bellorin et al. 2004), the North American coast in the Western Atlantic (Freshwater et al. 2006; Thomsen et al. 2006) as well as the European coast at the Eastern Atlantic, extending towards northern parts of the North Sea and the South Western Baltic Sea (Rueness 2005; Thomsen 2007; Weinberger et al. 2008). Epibiota associated with *G. vermiculophylla* have been studied across the Northern Hemisphere (Bonthond et al. 2020), which revealed that some epibiota and endobiota were part of a core, that is, a group of microbial taxa that was associated with the *G. vermiculophylla* holobiont irrespective of the host geography. This holobiont was also studied in controlled experiments in the laboratory, and its microbiota were sampled repeatedly over several weeks to months (Bonthond et al. 2021a, 2023). These time series demonstrated that epibiota and endobiota within the holobiont have strong temporal variation and that not all geographic core microbiota were temporally stable. Altogether, this may indicate that many core microbes may be rather season‐specific. Given the wide geographic stretch, across which spatial variability and core microbes have already been studied in Bonthond et al. (2020), *G. vermiculophylla* presents a suitable seaweed holobiont to characterise seasonal variability and core microbiota.

The aim of the present study was therefore to evaluate seasonal variability in *G. vermiculophylla*‐associated epibiota as well as to characterise cyclic patterns and permanent core microbiota (i.e., seasonal and interannual). To this end, we conducted a time series sampling of *G. vermiculophylla*, sampling individuals every 2 months from two distinct populations over three consecutive years, resulting in a dataset with 18 repeated measures. We hypothesised that prokaryotic epibiota associated with *G. vermiculophylla* show seasonality, in terms of taxonomic and functional composition and in terms of diversity. Furthermore, we hypothesised that *G. vermiculophylla* harbours core microbiota, which are (i) permanent (i.e., associated irrespective of time and space) as well as (ii) season‐specific (i.e., consistently present in the holobiont during specific times of the year).

## 2 Experimental Procedures

### 2.1 Sample Collection

**FIGURE 1:**
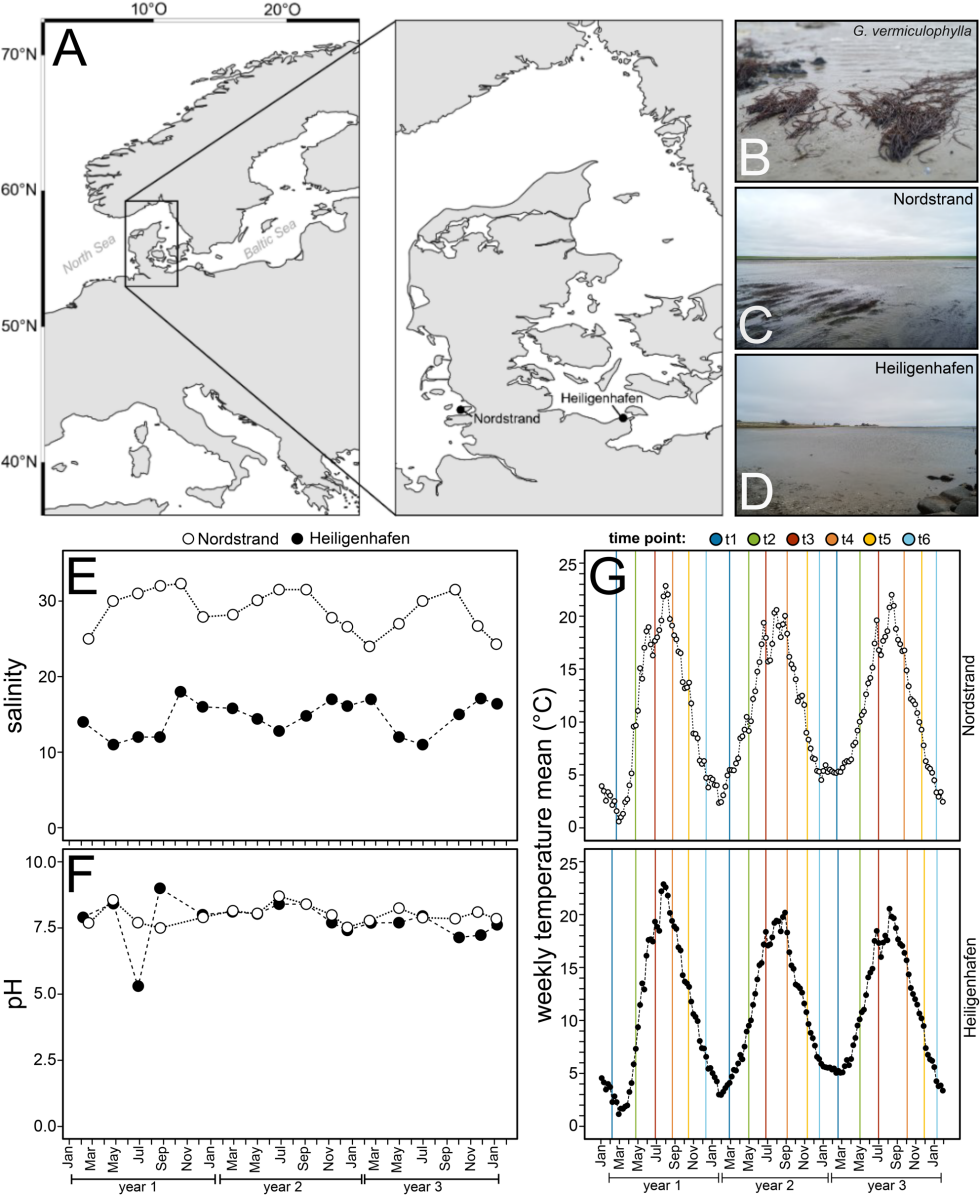
Overview of the two collection sites Nordstrand (North Sea) and Heiligenhafen (Baltic Sea), where the two‐month sampling of *Gracilaria vermiculophylla* was carried out, and environmental parameters during the three‐year time period. (A) Map showing the two collection sites. (B) Habitus of *G. vermiculophylla*. (C) North Sea population found at Nordstrand, (D) Baltic Sea population found at Heiligenhafen. (E) Salinity and (F) pH measured during the field sampling. (G) The weekly water temperature (°C) obtained from nearby measuring stations, with vertical lines depicting the exact collection time points. Temperature data was provided by the German Federal Maritime and Hydrographic Agency (BSH 2023).

Seaweeds were collected in Nordstrand (Germany) at the North Sea (54°29′9.34″N 8°48′44.65″E) and in Heiligenhafen (Germany) at the Baltic Sea (54°22′46.7″N 10°58′57.5″E; Figure 1A–D). These populations were chosen based on their distinct environmental features. The North Sea population is found in the intertidal zone. Here, the perennial *G. vermiculophylla* occurs mainly attached to hard substratum and can build massive mats during spring and/or summer. In contrast, the Baltic Sea population is situated in a small lagoon sheltered from turbulences and is only experiencing wind‐driven sea‐level fluctuations. Here, the *G. vermiculophylla* individuals are not attached to the substratum but rather loosely embedded in the soft sediment. In contrast to the North Sea population, the population at the Baltic site typically reduces substantially during winter and sometimes appears to be absent. Whereas individuals in the North Sea population are exposed to fully marine salinities (i.e., ~24–32, Figure 1E) and diurnal air exposure, the Baltic Sea population experiences rather brackish salinities (between ~10 and 20, Figure 1E) as well as less and irregular air exposure. Generally, the pH is more similar between the two populations, normally fluctuating between 7 and 9, although an outlier was detected in Heiligenhafen of 5.3, recorded during early summer in year 1 (Figure 1F).

The sample collection took place in two‐month intervals covering a three‐year time period from February 2018 to January 2021. During these years, water temperatures at locations nearby to Nordstrand and Heiligenhafen oscillated similarly between minima of 1 to 6°C in winter and maxima of 21 to 23°C in summer (Figure 1G). At each sampling point, 10 *G. vermiculophylla* individuals were collected with gloves and placed separately into plastic bags. To avoid collection of the same individual, the individuals were sampled at least 1 m away from each other. In the North Sea population, only attached algae were sampled. Additionally, three 50‐mL water and two 15‐mL sediment samples were taken at both sites. Subsequently, around 0.25‐mL sediment was transferred into a 2‐mL tube containing absolute ethanol. After collection, all samples were transported in a cooling box back to the facilities of GEOMAR Helmholtz Centre for Ocean Research in Kiel (Germany) where they were stored at 4°C to preserve the tissue integrity, after which the extraction procedure to separate epiphytes from the host tissue was carried out as soon as possible and at least within 48 h.

In the laboratory, salinity and pH were measured for both collection sites from one of the three water samples. The remaining water samples were processed further together with the seaweed samples.

### 2.2 Generating Epiphytic Extracts

To generate extracts from the prokaryotic epibiota associated with *G. vermiculophylla*, the method in Bonthond et al. (2020) was followed. In brief, an apical branch (i.e., representing the younger tissue) of 1 ± 0.25 g was transferred into a 50‐mL tube. Approximately 10 glass beads and 15‐mL artificial seawater of the respective salinity (prepared from distilled water and sodium chloride) were added. Besides the field samples, at least one blank was prepared for each sampling event, containing only glass beads and distilled water. Then, samples were vortexed for 2 min at maximum rotation speed. After vortexing, the algal tissue was removed and the suspension of epiphytes was used for DNA extraction. For the samples collected in the first sampling year, the epiphytic suspension was filtered as in Bonthond et al. (2020). During the second and third years, the centrifugation method of Ficetola et al. (2008) was used. In brief, 33 mL of absolute ethanol and 1.5 mL of sodium acetate were added to 15 mL epiphytic suspension, water and blank samples. All tubes were mixed and either processed immediately or cooled at 4°C and processed in the next days. The 50‐mL tubes were then centrifuged for 10 min at 14,000*g*. The supernatant was discarded and the pellet was preserved in 1 mL absolute ethanol. The generated epiphytic algal extracts (on filters or in 1 mL ethanol), water, sediment, and blanks were stored at −20°C until DNA extraction.

### 2.3 DNA Extraction and Amplicon Library Preparation

Ethanol was removed from the samples by evaporation in a vacuum centrifuge for at least 1 h at 45°C. If the evaporation of the alcohol was unsuccessful after several hours, the remaining ethanol was removed by lyophilisation. Filters were fragmented to small pieces with sterile scissors. Subsequently, DNA was extracted following the cetyltrimethylammonium bromide (CTAB)–chloroform protocol from Doyle and Doyle (1987). The amplicon library was prepared following a two‐step PCR approach by Gohl et al. (2016), using the same indexing primers and KAPA HIFI HotStart polymerase (Roche). The first PCR targeted the V4 region of the 16S rRNA gene using the forward primer U515F (S‐*‐Univ‐0515‐a‐S‐19) and the reverse primer 806R (S‐D‐Arch‐0786‐a‐A‐20; Klindworth et al. 2013) with adapters for the second PCR on 5′ ends. The first PCR programme began with a step of 5 min at 95°C and was followed by 25 cycles of denaturation for 20 s at 98°C, annealing for 15 s at 55°C and elongation for 1 min at 72°C. For water samples which were not successfully amplified, 30 cycles were used in a repeated PCR attempt.

For the second PCR, the amplicon products were diluted 1:10 and used as template. PCR was conducted following the same cycling programme but with 10 cycles of denaturation and an additional final elongation step of 10 min at 72°C. Subsequently, PCR products were visualised by gel electrophoresis and relative amplicon concentrations were estimated from gel pictures using the software Image J Fiji (Fiji for Mac OS X Version 1.0) to accordingly adjust volumes in the library pooling. The pooled library was purified with a gel extraction step by using the ZymoClean Gel DNA recovery kit (ZymoResearch) following the supplied protocol, quantified with qPCR and sequenced as paired‐end reads (2 × 300) on the Illumina MiSeq platform at the Max Planck Institute in Plön (Germany).

### 2.4 Data Processing

A total of 406 samples (including controls) were processed with the software Mothur (v.1.43.0 and v.1.45.3; Schloss et al. 2009) following an in‐house script. Accordingly, unique sequences were aligned to and classified with the SILVA reference alignment v132 (Quast et al. 2013). The sequences were open‐reference clustered to the 3% OTUs from a field study by Bonthond et al. (2020) with the *cluster. fit()* function (Sovacool et al. 2022). Sequences of mitochondrial, chloroplast, eukaryotic and unknown origin were removed. Finally, we removed OTUs that were singletons in the full dataset and samples that were considered to have low coverage (< 1000 reads). The raw demultiplexed amplicon reads were deposited in the SRA database (accession: PRJNA1155875). Predicted metagenomes based on KEGG Ortholog (KO) annotations (Kanehisa et al. 2014) were obtained with PICRUST2 v2.5.0 (Douglas et al. 2020).

The variability in the taxonomic (based on OTUs) and functional (based on predicted KOs) community composition over time was visualised with nonmetric multidimensional plots (nMDS) using Bray‐Curtis distances with the R package vegan (Oksanen 2010), based on rarefied data of either all samples including all substrates (alga, water and sediment) or solely algal substrates only. Additionally, trajectories were drawn chronologically through the group centroids of algal samples from the same sampling event. To test for differences in taxonomic and potential functional community composition, permutational multivariate analysis of variance (PERMANOVA) was applied on the unrarefied OTU dataset, by using 9999 permutations in the R package vegan (Oksanen 2010; usage of the adonis2 function). First, a PERMANOVA was run on the full OTU and KO datasets, including alga, water and sediment samples. The model included the variables season (as factor with 6 levels), year (as factor with 3 levels), population (as factor with 2 levels), substrate (alga, water and sediment) and all interactions. Subsequently, a PERMANOVA was run on the OTU and KO datasets of only algal samples, with the variables season, year, population and all possible interactions. In both PERMANOVAs, the log transformed sequencing depth (LSD) was included as covariate to account for differences in sequencing depth across samples.

To analyse the microbial diversity, the OTU richness was calculated with the R package iNEXT v3.0.0 (Hsieh et al. 2016). Second, as a measure of evenness, the probability of interspecific encounter (PIE; Hurlbert 1971) was calculated with the R package mobr v2.0.2 (McGlinn et al. 2019). For both diversity measures, the total datasets containing exclusively algal OTU read counts were used. For richness, a generalised linear model (GLM) was fitted as a function of season (as a factor with 6 levels, corresponding to the sampling events repeated over 3 years), year (as a factor with 3 levels), population (as a factor with 2 levels) and all possible interactions. The model assumed a Gaussian family distribution, with a logarithm in the link function. The LSD was included as a covariate to account for variation in total read counts across samples. For the PIE, a GLM with the same model structure was used. PIE was logit transformed to meet the model assumptions. As the LSD was not significant, it was excluded from the model. Analysis of variance (ANOVA) was applied to the GLM of richness and PIE to test for significance. If the main effects of season, year and population were significant, post hoc analysis was performed by pair‐wise comparisons in both models on all possible interactions between the main effects by using the R package emmeans v1.8.9 (Lenth 2022).

### 2.5 Defining Permanent and Seasonal Core Microbiota

Core microbiota were identified using two alternative compositional approaches by identifying differentially abundant OTUs (see Shade and Handelsman 2012 for definitions of core microbiomes). With both approaches, we defined both a permanent core, including OTUs persistently detected within the epibiota across all seasons and years, and two seasonal cores, including OTUs consistently detected within either summer or winter.

The first approach was based on multivariate GLMs (mGLMs) from the R package mvabund v4.2.1 (Wang et al. 2012), which was also used to characterise the spatial core in Bonthond et al. (2020). mGLMs were fitted on the cumulative 95% most abundant OTUs with > 25% prevalence and assumed a negative binomial distribution. For the permanent core, the variables substrate (alga, water or sediment), season (six levels, *t*_1_: *t*_6_) and year (three levels) were included as predictors. For seasonal mGLM cores, the OTU matrix was reduced to season time points *t*_1_ (late winter), *t*_3_ (early summer), *t*_4_ (late summer) and *t*_6_ (early winter) and the factor ‘season’ was reduced to only two levels representing the seasonal extremes (winter: *t*_6_ and *t*_1_, summer: *t*_3_ and *t*_4_), and included in the model together with the factor year. Both mGLMs included the LSD as offset to correct for different sequencing depths across samples. Models were resampled using the summary.manyglm function with 500 bootstrap iterations, which were restricted within populations. The *p*‐values were obtained through Wald tests. OTUs were considered part of the permanent core when the coefficients substrate_alga:water_ and substrate_alga:sediment_ were negative (reflecting higher relative OTU abundances associated with algal samples compared to water and sediment) and with corresponding *p*‐values < 0.01. Similarly, OTUs with positive and negative coefficients for the factor season_summer:winter_ and with *p*‐values < 0.01, were considered winter and summer core OTUs, respectively.

In addition to the mGLM core, we also defined a compositional core with the linear discriminant analysis effect size method (LEfSe, Segata et al. 2011) through the online interface at the webpage from the Huttenhower lab (https://huttenhower.sph.harvard.edu/galaxy/; accessed May 2023). For the permanent LEfSe core, OTUs significantly more abundant in epibiota samples compared to both water and sediment samples were considered core OTUs. Also, for seasonal LEfSe cores, the dataset was reduced to summer (time points *t*_3_ and *t*_4_ combined) and winter (time points *t*_1_ and *t*_6_ combined) to identify OTUs significantly more abundant in either season.

## 3 Results

### 3.1 Sequencing Summary

After all quality filtration steps, the final dataset counted 262 samples (170 algal, 29 water and 63 sediment samples) and 14,874,961 sequencing reads, clustered into 45,751 OTUs, including 17,670 OTUs that were already identified in Bonthond et al. (2021a, 2021b). Due to the quality filtering process, a few replicates were not included in downstream analyses. However, with the exception of one group with two replicates, one group with three replicates and five groups with four replicates, five replicates remained for each population at each timepoint (29 out of 36 groups).

**FIGURE 2:**
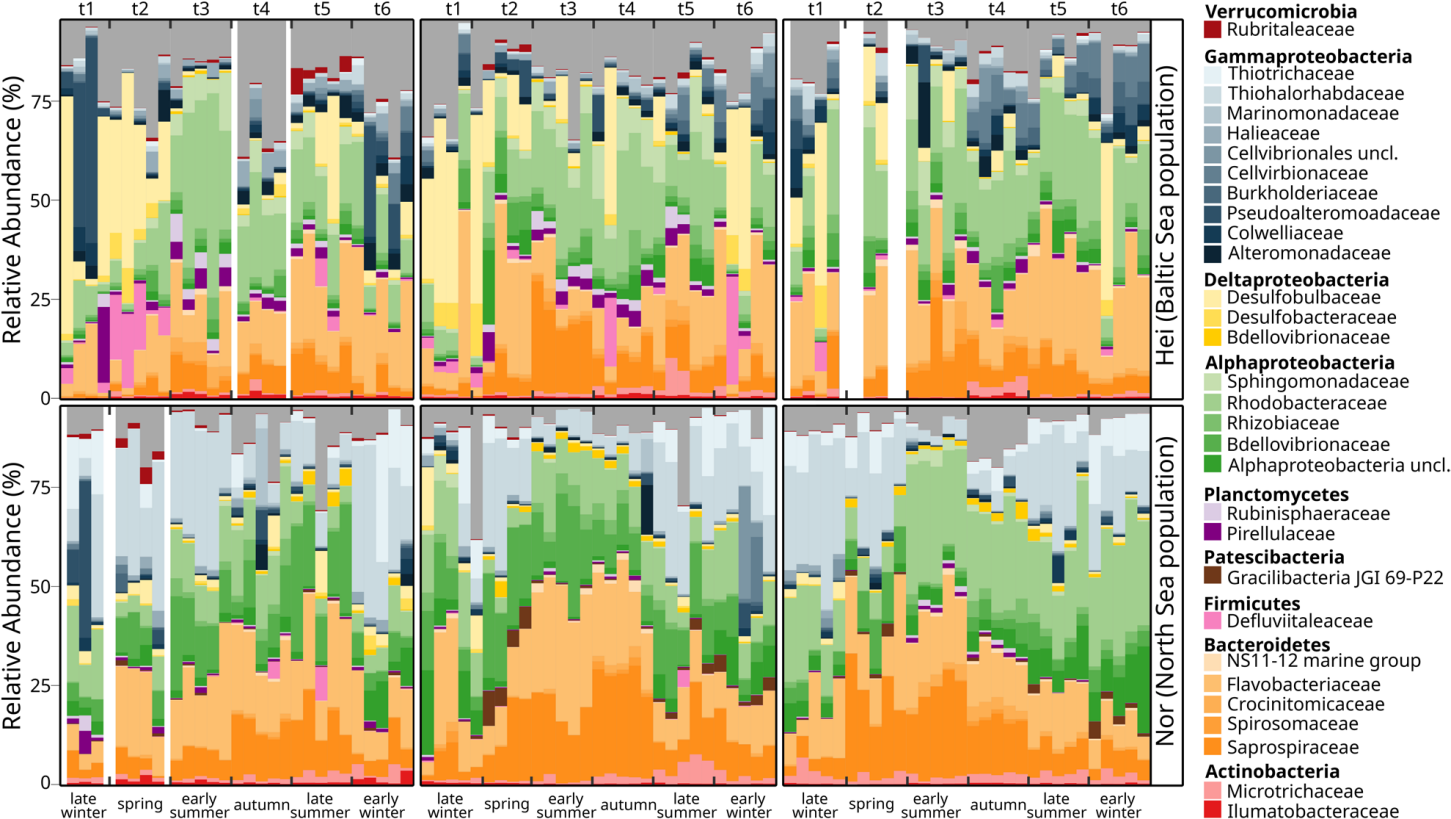
Microbial community composition of the 30 most abundant families associated with the surface of the red seaweed *Gracilaria vermiculophylla* in mean relative abundance (%). Columns represent individual replicates and are ordered by population, year and season.

The overall most abundant OTU in the holobiont was classified to *Granulosicoccus* (OTU97) with a mean relative abundance of 4.93% and 99.4% occupancy, followed by OTUs classified to Alphaproteobacteria (OTU12, 1.82% abundance and 100% occupancy), Rhodobacteraceae (OTU3, 1.73% relative abundance and 100% occupancy) and *Desulforhopalus* (OTU1577, 1.51% relative abundance and 91.60% occupancy). The most abundant families were Rhodobacteraceae (18.17%), Flavobacteriaceae (15.13%), Saprospiraceae (10.73%) and Thiohalorhabdaceae (4.81%, Figure 2).

The prokaryotic surface communities associated with the red seaweed *G. vermiculophylla* compositionally differed from the seawater and sediment communities. Although the classes Gammaproteobacteria, Alphaproteobacteria, Bacteroidia and Deltaproteobacteria contributed to the general community composition structure and were shared between all three substrates, detectable differences in abundance occurred already in the top 10 families (Figure S1). Notably, the 10 most abundant prokaryotic families contributed around 65% of all families present on *G. vermiculophylla* but less than 50% of those were present in sediment and water. The families Flavobacteriaceae and Rhodobacteraceae were particularly important in algal and seawater samples, while Halieaceae, Desulfobulbaceae, Desulfobacteraceae, Chromatiaceae and Pirellulaceae were more important in sediment samples. Interestingly, Thiohalorhabdaceae, Thiotrichaceae, Sphingomonadaceae and Pseudoalteromonadaceae only occurred in the top 10 families of algal samples (Figure S1).

Microbiota on algal surfaces varied considerably over time. Regarded for both populations together and throughout the years, Gammaproteobacteria were mainly represented by Thiohalorhabdaceae, Alphaproteobacteria by Rhodobacteraceae, and Bacteroidia by Flavobacteriaceae and Saprospiraceae. This pattern was best visible in the second and third years (Figures 2 and S2). Additionally, each year had a specific pattern of interchanging families along seasons. Desulfobulbaceae tended to be less prominent in summer than in winter, while an opposite pattern was observed for Rhizobiaceae (Figures 2 and S3). However, exceptions occurred. For instance, Rhizobiaceae were also relatively abundant in winter (*t*_6_) in the third year and an exceedingly high abundance of Pseudoalteromonadaceae was once observed at *t*_1_ at the beginning of the first year.

Epiphytic communities also varied between the populations (Figure S2). Thiohalorhabdaceae, Thiotrichaceae, Microtrichaceae and Rhizobiaceae solely occurred in the 10 most abundant families in the North Sea population, whereas dominant families associated primarily with the Baltic Sea population were Pseudoalteromonadaceae, Sphingomonadaceae, Cellvibrionaceae and Pirellulaceae. A comparison of community compositions between the two populations over time (Figure 2) generally confirmed these differences.

### 3.2 Community Composition

An nMDS based on taxonomic composition clearly separated algal samples and sediment samples, while seawater samples were arranged amid those two (Figure S4). A PERMANOVA confirmed that much compositional variation was explained by the substrate (*R* ^2^ = 0.117; *p* < 0.001, Table S1). Among algal samples, microbial taxonomic composition varied between populations (*R* ^2^ = 0.109; *p* < 0.001; Table S2). nMDS correspondingly separated samples collected from algal surfaces and sediment samples from the North and Baltic Sea populations nearly completely, while water and sediment samples exhibited more compositional similarity between populations (Figure S4).

**FIGURE 3:**
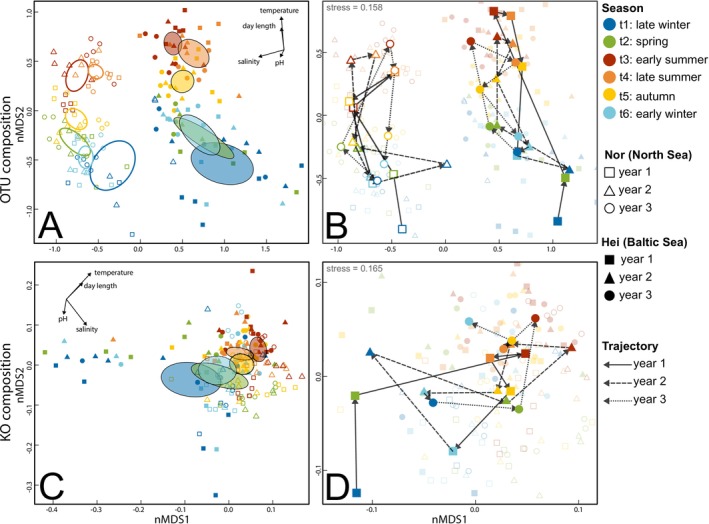
Nonmetric multidimensional scaling (nMDS) of the microbial taxonomic (A, B) and functional (C, D) composition associated with *Gracilaria vermiculophylla*. (A, C) The corresponding ellipses show the group centroids of each timepoint and the corresponding 95% confidence region, in (A) separately for the North and Baltic Sea populations and in (C) for both populations together. The abiotic variables temperature, day length, salinity and pH are ordinated on the nMDS plots and shown in black arrows. (B, D) Zoomed in panels of the nMDS plots in (A) and (C), respectively, with trajectories drawn chronologically through the centroids of all 18 timepoints. Stress values are given in the upper left corners.

Microbial taxonomic community composition varied also significantly over time. Season and year together (individually or in interaction with other factors) importantly explained compositional differences between samples (PERMANOVA, Table S1 for all samples and Table S2 for algal samples only). The nMDS also indicated that the taxonomic composition of epiphytic communities varied strongly by season, with similar seasonal shifts across years (Figure 3A). Correspondingly, the PERMANOVA confirmed that the clustering by season (*R* ^2^ = 0.092; *p* < 0.001; Table S2) was stronger than clustering by year (*R* ^2^ = 0.028; *p* < 0.001; Table S2). Shifts between winter and summer were similar between years (Figure 2B). Despite substantial differences between the two populations, the seasonal changes in microbial composition were similar in direction and magnitude.

While functional community composition was also strongly shaped by season (*R* ^2^ = 0.092; *p* < 0.001), differences between populations were limited (*R* ^2^ = 0.007, *p* = 0.041) and not clearly visible in the nMDS. Also, the year explained more functional diversity (*R* ^2^ = 0.016; *p* = 0.008) than the factor population, which was not the case for taxonomic diversity (Tables S2 and S3). Also for the functional composition, the nMDS revealed a strong transition from winter to summer and back, which was similar between years (Figure 3C,D).

### 3.3 Diversity

**FIGURE 4:**
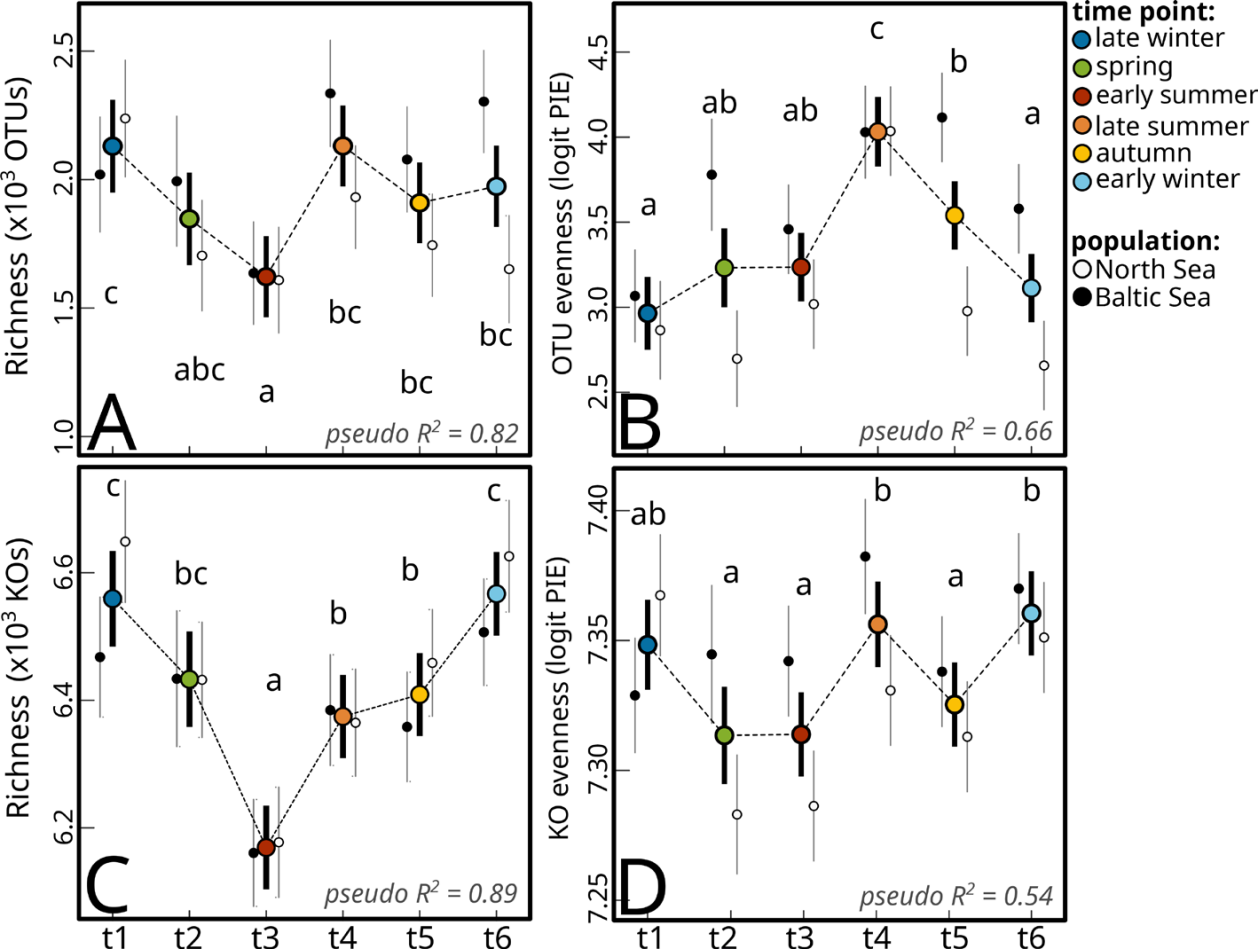
Estimated means of OTU richness (A), OTU evenness (B), KO richness (C) and KO evenness (D) from GLMs fitted on two diversity measures among the six season time points. Error bars show 95% confidence intervals. The Cox and Snell pseudo R^2^ is given in each corner of the respective plot. Significantly different time points within pair‐wise comparisons in the post hoc analysis are indicated by small letters.

The OTU richness of microbial communities on the surface of *G. vermiculophylla* showed significant seasonal variation (LR *χ* ^2^(5) = 37.331, *p* < 0.001; Table S4A). It was characterised by a minimum in early summer (*t*_3_) and a maximum in late winter (*t*_1_) and was rather constant from summer (*t*_4_) to early winter (*t*_6_; Figure 4A; Table S4B). The Baltic Sea population yielded higher OTU richness (LR *χ* ^2^(1) = 18.565, *p* < 0.001; Table S4A), especially during late summer towards early winter. A different seasonal pattern emerged for evenness (LR *χ* ^2^(5) = 86.574, *p* < 0.001; Table S5). PIE was highest in late summer (*t*_4_) and minimal during late winter (*t*_6_, Figure 4B). Evenness was also higher in the Baltic Sea population (LR *χ* ^2^(1) = 86.574, *p* < 0.001; Table S5A), where it reached its maximum during autumn. Similar to OTU richness, the richness of predicted KOs showed strong seasonal variation (LR *χ* ^2^(5) = 112.80, *p* < 0.001; Table S4A), with a maximum in late winter (*t*_1_) and minimum in early summer (*t*_3_), but transitioned more gradually towards these extremes over the other seasonal time points (Figure 4C, Table S4). In contrast with OTU richness, KO richness was slightly higher in the North Sea population (LR *χ* ^2^(5) = 7.12, *p* = 0.008; Table S4A). Evenness in predicted functions varied with season as well (LR *χ* ^2^(5) = 39.519, *p* < 0.001; Table S5A) but yielded a more complex trend with multiple optima (*t*_4_, *t*_6_) and minima (*t*_2_, *t*_3_, *t*_5_, Figure 4D, Table S5) and was highest in the Baltic Sea population (LR *χ* ^2^(1) = 19.463, *p* < 0.001; Table S5A).

### 3.4 Core Microbiota

**FIGURE 5:**
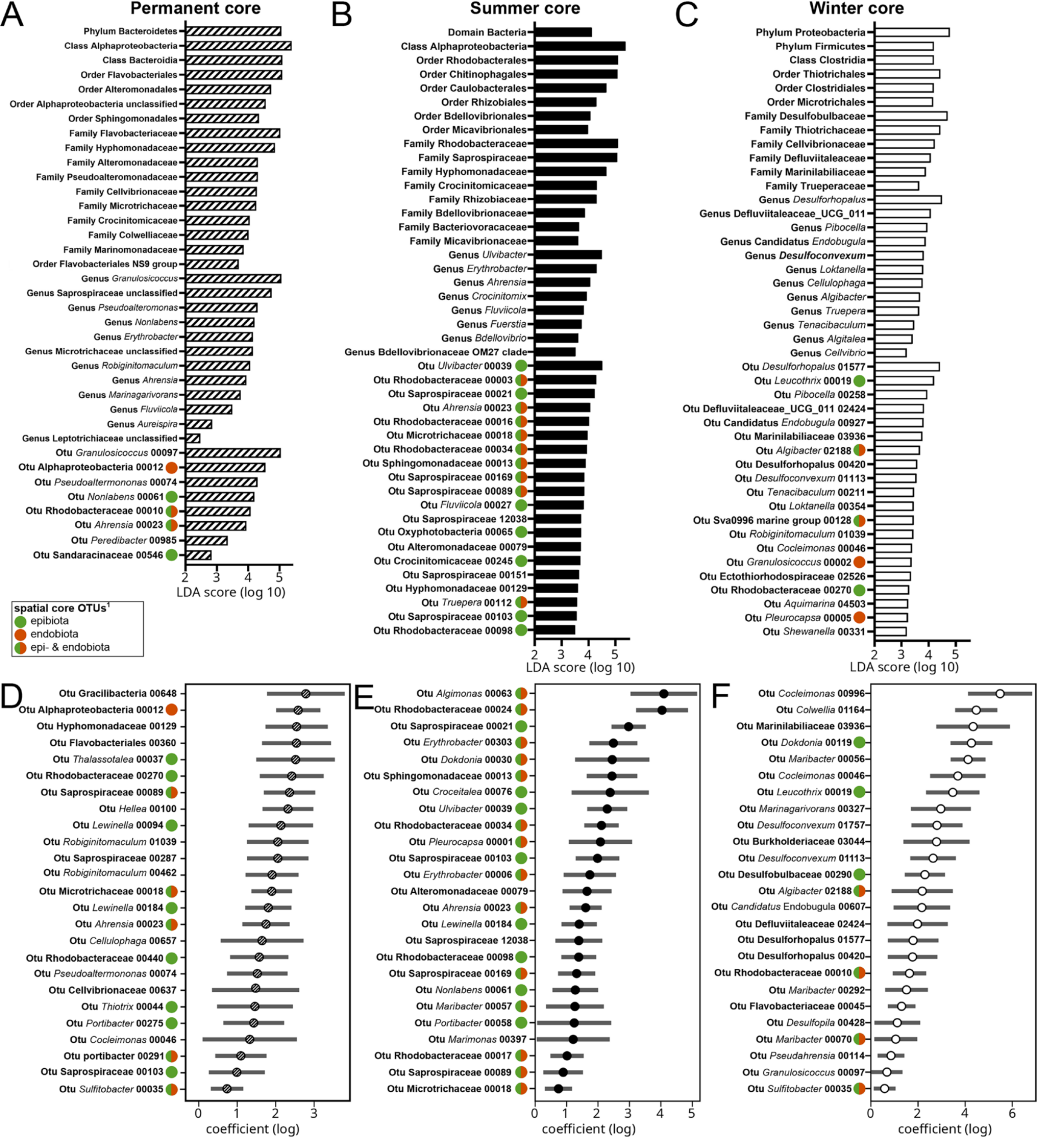
Permanent (A, D), summer (B, E) and winter (C, F) core epibiota associated with the rodophyte *Gracilaria vermiculophylla*. (A–C) Core taxa at different taxonomic levels detected with LEfSe. (D–F) Core OTUs detected using mGLMs. Only the top 25 most abundant core OTUs are shown. ^(1)^ Green, red and green‐red circles in front of the taxon labels indicate OTUs identified as spatial core OTUs in Bonthond et al. (2020).

Linear discriminant analysis identified several taxa as permanent biomarkers of *G. vermiculophylla* surfaces, that is, they were at all times of the year significantly less characteristic for sediment and water samples (Figure 5A, Tables S6 and S7). The approach identified 8 OTUs that formed a permanent LEfSe core of the algal host (Figure 5A, Table S6) and also several higher taxonomic ranks as LEfSe core groups (Table S7), including the phylum Bacteroidetes, the classes Alphaproteobacteria and Bacteroidea, four different orders (highest LDA score: Flavobacteriales), nine different families (highest LDA scores: Flavobacteriaceae and Hyphomonadaceae) and 12 different genera (Highest LDA score: *Granulosicoccus*). Altogether 69 OTUs formed a summer LEfSe core of *G. vermiculophylla* and 33 OTUs formed a winter LEfSe core (Figure 5B,C, Table S6). Biomarkers of summer (Figure 5B, Table S7) were the dominant bacteria, the class Alphaproteobacteria, six orders (highest LDA scores: Rhodobacterales and Chitinophagales), eight families (highest LDA scores: Rhodobacteraceae and Saprospiraceae) and eight genera (highest LDA score: *Ulvibacter*). Taxonomic biomarkers of the winter season (Figure 5C, Table S7) were the phyla Proteobacteria and Firmicutes, the class Clostridia, three orders (highest LDA score: Thiotrichales), six families (highest LDA score: Desulfobulbaceae) and 12 genera (highest LDA score: *Desulforhopalus*).

The permanent and seasonal cores defined by the mGLM approach overlapped to some extent with the cores characterised by the linear discriminant analysis. The permanent mGLM core counted 88 OTUs (Figure 5D for the 25 most abundant OTUs, Table S6). Five of the 8 OTUs LEfSe core OTUs were also identified as part of the mGLM core (Table S6). The summer mGLM core of *G. vermiculophylla* counted 205 OTUs (Figure 5E for the 25 most abundant OTUs, Table S6). Fifty of the 69 the LEfSe summer core OTUs were also part of the summer mGLM core. The mGLM winter core counted 285 OTUs. Twenty‐one out of the 33 LEfSe winter core OTUs were also part of the winter mGLM core (Figure 5F for the 25 most abundant OTUs, Table S6).

## 4 Discussion

This study revealed that epibiota associated with the seaweed holobiont *G. vermiculophylla* show strong seasonality. Prokaryotic composition and diversity are highly variable throughout the year. This variation is largely cyclic, showing similar trends over the three repetitive years in this study, with late winter and early summer as the extremes between which composition and diversity oscillate (Figures 3 and 4). Our data also indicate that these seasonal shifts are not functionally redundant, as concurrent trends were found in terms of composition and diversity of predicted functions. Therewith, these findings support our hypothesis that the *G. vermiculophylla* holobiont has seasonal dynamics, providing evidence that seaweed‐associated epibiota undergo seasonal successional cycles. In line with this, we found numerous seasonal core taxa, that is, microbial OTUs or groups of higher taxonomic ranks, that were consistently associated with either summer or winter. In addition, our study identified a permanent core, of microbial taxa which were consistently associated with the host independent of season.

### 4.1 Temporal Variation in Epibiota Is Highly Seasonal

Seasonal patterns have been described in seaweeds before, including chemical host processes such as metabolite production (Paix et al. 2019) or antifouling activity (Saha and Wahl 2013; Wang et al. 2018). Also in microbial communities associated with seaweeds, variability associated with seasonal changes has been observed (Bengtsson et al. 2010; Burgunter‐Delamare et al. 2023; Korlević et al. 2021; Lachnit et al. 2011; Mancuso et al. 2016; Park et al. 2022; Serebryakova et al. 2018; Tujula et al. 2010). However, while covering different seasons, the sampling in these studies is typically limited to 1 year (with the exception of Lachnit et al. 2011). Our findings are strongly in line with their observations, showing that also in the *G. vermiculophylla* holobiont, composition and diversity shift from one season to another. By repeating the seasonal sampling across three subsequent years, our study also resolves interannual trends which demonstrate that much of this temporal variation is repetitive and therefore truly seasonal (Fuhrman et al. 2015). Epibiota associated with *G. vermiculophylla* are thus highly dynamic but also resilient, as they undergo strong compositional shifts, but shift back towards compositions experienced in preceding years, in the same season.

Given the known structuring effects of both salinity (Stratil et al. 2014; Van Der Loos et al. 2023) and temperature on seaweed‐associated microbiota (Bonthond et al. 2023; Düsedau et al. 2023; Stratil et al. 2013), such seasonal environmental variables may well explain much of the here observed cyclic compositional and diversity changes. At the same time, they may also explain the pronounced differences in taxonomic composition that were observed between two sampling sites in the present study. The substantial taxonomic differences between the two populations are in line with previous research, wherein epiphytic communities were compared among 14 *G. vermiculophylla* populations, collected across the host distribution range on the Northern Hemisphere (Bonthond et al. 2020). The study showed that geographic variation in microbial epibiota is partially explained by differences between native and non‐native distribution ranges, but that there is also strong variation at the local scale. While we show here again that epibiota are compositionally different between populations, our work demonstrates at the same time that both populations change in comparable directions throughout the year.

Besides the environment, also the host undergoes metabolic, physiological and reproductive changes which can be season dependent (Liu et al. 2017 and references therein). Cycles in the host can also coincide with microbial life cycles, such as, for example, in the brown alga *Ascophyllum nodosum* , whose reproductive cycles are synchronised with the fungal symbiont *Stigmidium ascophylli* (Stanley 1992) or in *Acrochaetium* (Rhodophyta), in which bacterial metabolites (N‐acyl‐homoserine‐lactones) regulate spore release (Weinberger et al. 2007). In this context, an interesting observation is that functional and taxonomic composition of the bacterial communities associated with *G. vermiculophylla* oscillated seasonally with similar intensity, whereas pronounced taxonomic differences between sites were hardly reflected by similar functional differences. Different *G. vermiculophylla* epibiota appear to be functionally similar between sites in a given season but are functionally different among seasons. This suggests that the holobiont acquires season‐specific microbial functions but is rather promiscuous to the microbes that provide them.

### 4.2 Seasonal Shifts in Diversity

The shift from an OTU richness maximum in late winter to a minimum in summer (Figure 4A), as well as an inverse pattern of evenness (Figure 4B) was consistent across both populations and shows similarity to a study on temporal dynamics on the epibiota associated with the brown seaweed *Cystoseira compressa* (Mancuso et al. 2016). Moreover, this cyclic trend in richness appeared to be even stronger in terms of predicted functions, which implies that seasonal changes in diversity are not functionally redundant, resulting in more diverse functions in winter in the associated epibiota. Hypothetically, a decrease in taxonomic and functional richness towards summer may be driven by rising temperatures and solar irradiance, with which metabolic rates increase (Clarke and Fraser 2004; Gillooly et al. 2001), and reinforce competition and extinction rates within the seaweed microbiota. If this is true, the seasonal diversity cycle in *G. vermiculophylla* may be a more general trend among seaweed holobionts. However, due to limited studies on seaweed holobionts including both seasonal and interannual samplings this remains to be evaluated in future studies, targeting different seaweeds.

### 4.3 The Core Microbiome of *G. vermiculophylla*

Microbial cores have been studied across holobionts (Ainsworth et al. 2015; Burke et al. 2011; Schmitt et al. 2012; Shade and Handelsman 2012). Characterising the core microbiota of a host, particularly over a large spatial or temporal scale (Shade and Handelsman 2012), provides an opportunity to detect patterns of stability and generality within a holobiont. After Bonthond et al. (2020) characterised a spatial core of epi‐ and endophytes within *G. vermiculophylla*, by sampling different populations of the host across the Northern Hemisphere, the present work builds forward on this by characterising temporal cores.

To identify core taxa of *G. vermiculophylla*, we utilised two compositional approaches (Shade and Handelsman 2012), resolving core OTUs based on statistically significant differential abundances. Whereas the LEfSe approach is designed for hierarchical group comparisons and has the advantage of analysing differential abundances simultaneously at different taxonomic ranks (Segata et al. 2011), mGLMs are more flexible as they are capable of accounting for multiple covariates (here; population identity, substrate, year and sequencing depth). Both approaches corroborate our hypotheses that the *G. vermiculophylla* holobiont harbours prokaryotic taxa with strong temporal consistency, either associated permanently (Figure 5A,D) or recurrently in summer (Figure 5B,E) or winter (Figure 5C,F).

Jointly, the spatial and temporal cores of Bonthond et al. (2020) and this study provide an elaborate, and to our knowledge unprecedented, imprint of the core microbiota within a seaweed holobiont. A subset of 32 OTUs of the permanent mGLM core was also identified as part of the spatial core in Bonthond et al. (2020). This set of OTUs is thus both geographically and temporally highly conserved, and represents a core that appears to be unconditionally present within this seaweed holobiont. In addition, 37 summer core OTUs and 50 winter core OTUs were identified as spatial core OTUs in the previous study. While their presence in the *G. vermiculophylla* holobiont is season‐specific, they are also spatially and temporally consistent holobiont members.

Each of these taxa is of special interest, as their prevalent signal is unlikely coincidental. Perhaps most striking is the unclassified Alphaproteobacterial OTU (OTU12), which occurrence is 100% in both studies and whose poorly resolved identity may also indicate that the microbe is highly host‐specific, and is difficult to isolate individually. Similarly, a member of the genus *Ahrensia* (OTU23), was present in 100% and > 99% of all samples in the spatial and present study, respectively. In addition to being identified as core spatial endophyte, both mGLM and LEfSe approaches resolved the *Ahrensia* OTU as permanent core member, implying a consistent presence in the holobiont.

The presence of *Maribacter* core OTUs could hint at a host–microbe relationship within the *G. vermiculophylla* holobiont, similar to the chlorophyte *Ulva*, in which this bacterium plays a regulatory role in host morphogenesis (Weiss et al. 2017). Furthermore, the summer core also included the cyanobacterial OTU (OTU1), classified to *Pleurocapsa*, that was the most abundant OTU in the spatial study of Bonthond et al. (2020) and which was there found to be part of both epi‐ and endophytic cores. A closely related OTU (OTU7, classified to *Pleurocapsa* as well), also identified as spatial core endophyte and one of the most abundant OTUs in Bonthond et al. (2020), was here resolved as permanent core member. These cyanobacterial *Pleurocapsa* OTUs are closely related to *Waterburya agarophytonicola*, which was isolated from the same host and has the genomic potential to synthesise various vitamins, including cobalamin (vitamin B_12_) for which *G. vermiculophylla* is auxotroph (Bonthond et al. 2021b). Such cyanobacterial core members may thus potentially play a role in vitamin acquisition for the seaweed host.

Noteworthy is the detection of three *Granulosicoccus* OTUs as part of the winter core (OTUs 2, 41 and 97), of which two were resolved as spatial core endophytes in Bonthond et al. (2020). The genus *Granulosicoccus* is considered to be a seaweed generalist and is often reported as core symbiont (Aires et al. 2023; Park et al. 2022). Metagenomic evidence from the Kelp *Nereocystis luetkeana* suggested that associated *Granulosicoccus* have diverse energy metabolism, but are incapable of autotrophic carbon fixation, which may indicate they obtain organic carbon from their seaweed host (Weigel et al. 2022). Moreover, Weigel et al. (2022) also found that *Granulosicoccus* have all genes necessary to synthesise cobalamin, which makes them another candidate vitamin source for the auxotrophic host *G. vermiculophylla*.

Our study shows that permanent, summer and winter cores in *G. vermiculophylla* include various microbial taxa. These phylogenetically diverse holobiont members can be presumed to have distinct traits and provide variable functions to their host. What these traits are and how they are relevant for the host is an important open question for future study. The predicted functions used in our study support that changes in microbial composition and diversity come with strong functional shifts. Therewith, this work merits the need for in‐depth analyses on microbial functions, using more robust methods, such as metagenomic sequencing, to elucidate the functional dynamics of seasonal microbial succession in *G. vermiculophylla* and other seaweed holobionts.

## 5 Conclusions

Altogether, this research provides to the best of our knowledge one of the most detailed studies on seasonality in microbiota within a seaweed holobiont, with sampling two populations every 2 months, over 3 years. Epibiota associated with *G. vermiculophylla* are dynamic, and seasonality drives much of this temporal variation in diversity and composition. These seasonal differences are likely linked to environmental conditions such as salinity and temperature, which fluctuate strongly throughout the year, especially in the shallow and intertidal habitats where this seaweed typically occurs. Despite strong compositional differences between North and Baltic Sea populations, similar cyclic patterns were resolved between the two populations, reflecting that despite strong differences between populations, they experience similar seasonal succession cycles, and which are thus likely natural to *G. vermiculophylla* holobionts. These succession cycles entail functional changes, as the cyclic trend was also evident in predicted functional composition. In contrast, differences between populations were minimal in terms of functional composition, suggesting that unlike the spatial shifts, seasonal changes are more functional. Based on this we posit that spatial variability in microbial composition within the *G. vermiculophylla* holobiont is more redundant than seasonal variability because essential microbial functions can be obtained from a wide range of microbes.

While epibiota vary in space and time, we resolved 32 OTUs, which are permanent core members in this study and part of the spatial core characterised in earlier work of Bonthond et al. (2020). Therewith, this study demonstrates that certain microbial taxa are perpetual within the holobiont and are season and geography independent. This spatial and temporal core presents a subset of candidate microorganisms that may play important roles in host functioning and which merits future attention.

## Author Contributions

**Chantal Marie Mudlaff:** investigation, writing – original draft, methodology, visualization, writing – review and editing, formal analysis, data curation, validation, conceptualization. **Florian Weinberger:** formal analysis, supervision, visualization, funding acquisition, writing – review and editing, writing – original draft, validation, conceptualization, investigation, methodology. **Luisa Düsedau:** investigation, writing – review and editing, methodology. **Marjan Ghotbi:** methodology, investigation, writing – review and editing. **Sven Künzel:** writing – review and editing, investigation, methodology. **Guido Bonthond:** conceptualization, investigation, writing – original draft, writing – review and editing, visualization, validation, supervision, data curation, methodology, formal analysis.

## Conflicts of Interest

The authors declare no conflicts of interest.

## Supporting information


**Figure S1.** Stacked bar plots showing community composition by substrate.
**Figure S2.** Stacked bar plots showing community composition by population.
**Figure S3.** Stacked bar plots showing community composition by season and year.
**Figure S4.** nMDS with algal, water and sediment samples.
**Table S1.** PERMANOVA community composition all substrates.
**Table S2.** PERMANOVA community composition on only algal substrate.
**Table S3.** PERMANOVA predicted functional composition on only algal substrate.
**Table S4.** (A) ANOVA table for richness based on OTUs and KOs. (B) Post hoc pair‐wise comparisons within the factor season (*t*_1_–*t*_6_) for richness based on OTUs and KOs.
**Table S5.** (A) ANOVA table for evenness (probability of interspecific encounter) based on OTUs and KOs. (B) Post hoc pair‐wise comparisons within the factor season (*t*_1_–*t*_6_) for evenness (probability of interspecific encounter) based on OTUs and KOs.

**Table S6.** Epiphytic cores.
**Table S7.** Higher taxonomic rank cores.

## Data Availability

The raw de‐multiplexed V4‐16S rDNA gene amplicon reads and associated metadata are available from the SRA database under the Bioproject accession number PRJNA1155875. Other data and R‐scripts for analyses are available on GitHub at https://github.com/gbonthond/Seasonalilty_seaweed_holobiont. Supporting Information files can also be accessed on the GitHub repository that is cited in the article (DOI: 10.5281/zenodo.14811508).
