# Supplementary material for: Seasonal Cycles in a Seaweed Holobiont: A Multiyear Time Series Reveals Repetitive Microbial Shifts and Core Taxa
Source: Environ Microbiol. 2025 Feb 27;27(3):e70062. doi: 10.1111/1462-2920.70062 (PMC11867712; doi:10.1111/1462-2920.70062)
Supplement: Supplementary file 1 — Figure S1. Stacked bar plots showing community composition by substrate. Figure S2. Stacked bar plots showing community composition by population. Figure S3. Stacked bar plots showing community composition by season and year. Figure S4. nMDS with algal, water and sediment samples. Table S1. PERMANOVA community composition all substrates. Table S2. PERMANOVA community composition on only algal substrate. Table S3. PERMANOVA predicted functional composition on only algal substrate. Table S4. (A) ANOVA table for richness based on OTUs and KOs. (B) Post hoc pair‐wise comparisons within the factor season (t 1–t 6) for richness based on OTUs and KOs. Table S5. (A) ANOVA table for evenness (probability of interspecific encounter) based on OTUs and KOs. (B) Post hoc pair‐wise comparisons within the factor season (t 1–t 6) for evenness (probability of interspecific encounter) based on OTUs and KOs. [file EMI-27-e70062-s002.pdf]

Supporting Information

Seasonal cycles in a seaweed holobiont: A multiyear time series reveals repetitive microbial shifts and core taxa

Chantal Marie Mudlaff[1,2], Florian Weinberger[1], Luisa Düsedau[1,3], Marjan Ghotbi[1,2], Sven Künzel[4], Guido Bonthond[1,5,*]

[1] Department of Marine Ecology, GEOMAR Helmholtz Centre for Ocean Research Kiel, 24105 Kiel, Germany
[2] Faculty of Mathematics and Natural Sciences, Christian-Albrechts-Universität zu Kiel, 24118 Kiel, Germany
[3] Section Benthic Ecology, Alfred Wegener Institute Helmholtz Centre for Polar and Marine Research, 27570 Bremerhaven, Germany
[4] Department of Evolutionary Genetics, Max Planck Institute for Evolutionary Biology, Plön, Germany
[5] Institute for Chemistry and Biology of the Marine Environment (ICBM), School of Mathematics and Science, Carl von Ossietzky Universität Oldenburg, Ammerländer Heerstraße 114-118, 26129 Oldenburg, Germany
* Corresponding author: guido.bonthond@uol.de

FIGURES

Figure S1. Stacked bar plots showing community composition by substrate
Figure S2. Stacked bar plots showing community composition by population
Figure S3. Stacked bar plots showing community composition by season and year
Figure S4. nMDS with sediment and water samples

TABLES

Table S1. PERMANOVA community composition all substrates
Table S2. PERMANOVA community composition on only algal
Table S3. PERMANOVA predicted functional composition
Table S4. Analysis of Deviance tables from diversity metrics analyzed with GLMs and likelihood ratio tests.
Table S5. Table S5. Post-hoc pairwise comparisons for all levels within the predictor season.
Table S6. Epiphytic cores (see separate Excel file)
Table S7. Higher taxonomic rank cores (see separate Excel file)

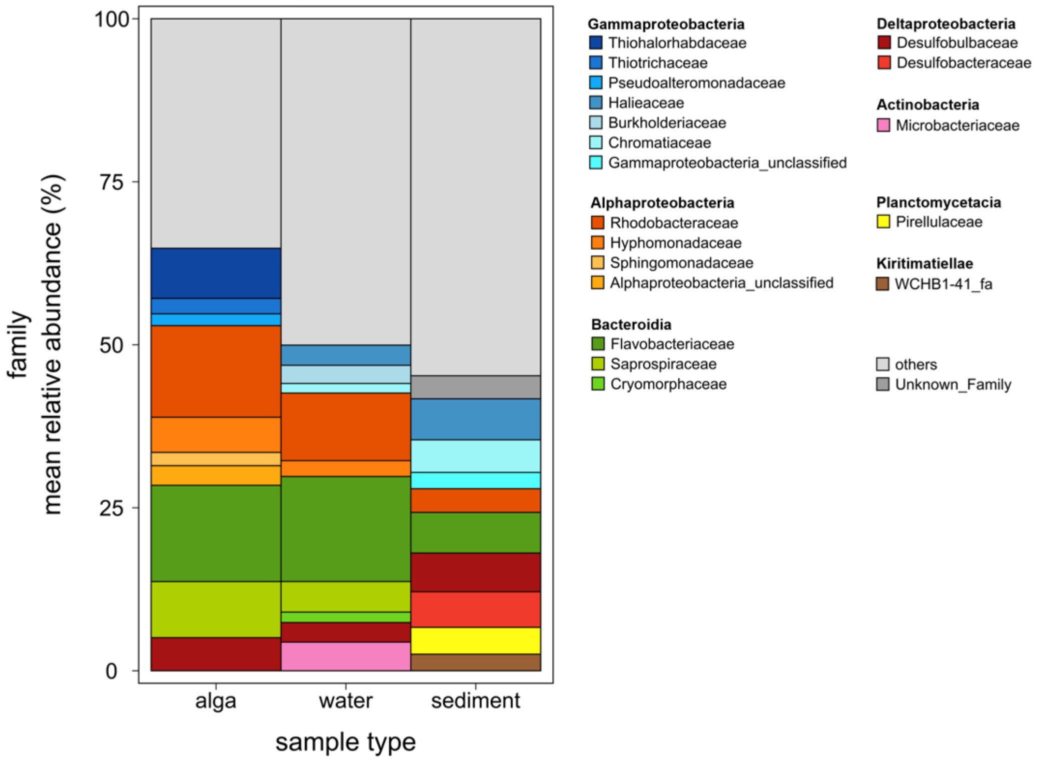

Figure S1. Microbial community composition of the ten most abundant families associated with the sample types alga, water and sediment. Shown is the mean relative abundance in percent (%). Main colours fit to the taxonomic class and corresponding families are illustrated with a variation of that main colour. The fraction “others” counts all families not represented in the ten most abundant members. Normalized data on total OTU read counts is shown.

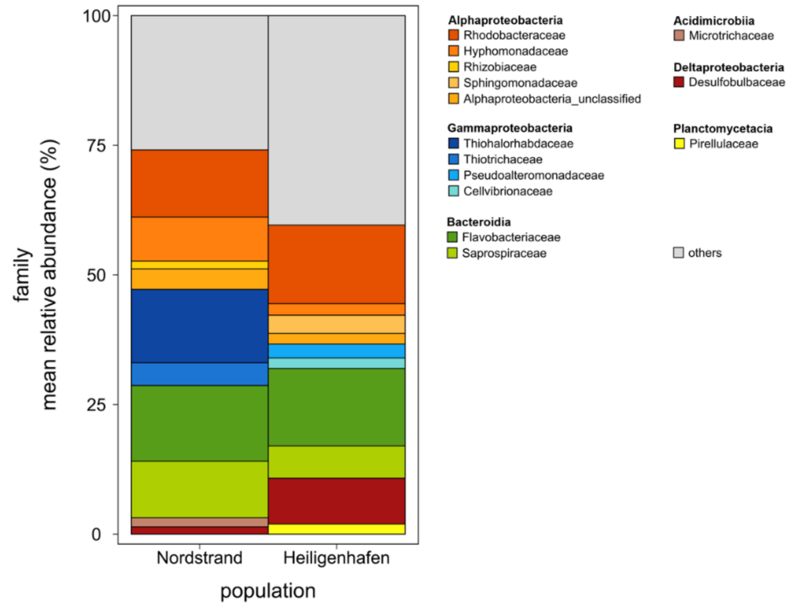

Figure S2. Microbial community composition of the ten most abundant families associated with the surface of the red seaweed *Gracilaria vermiculophylla* divided between the two populations Nordstrand (North Sea) and Heiligenhafen (Baltic Sea). Shown is the mean relative abundance in percent (%). Main colours fit to the taxonomic class and corresponding families are illustrated with a variation of that main colour. The fraction “others” counts all families not represented in the ten most abundant members. Normalized data on total OTU read counts is shown.

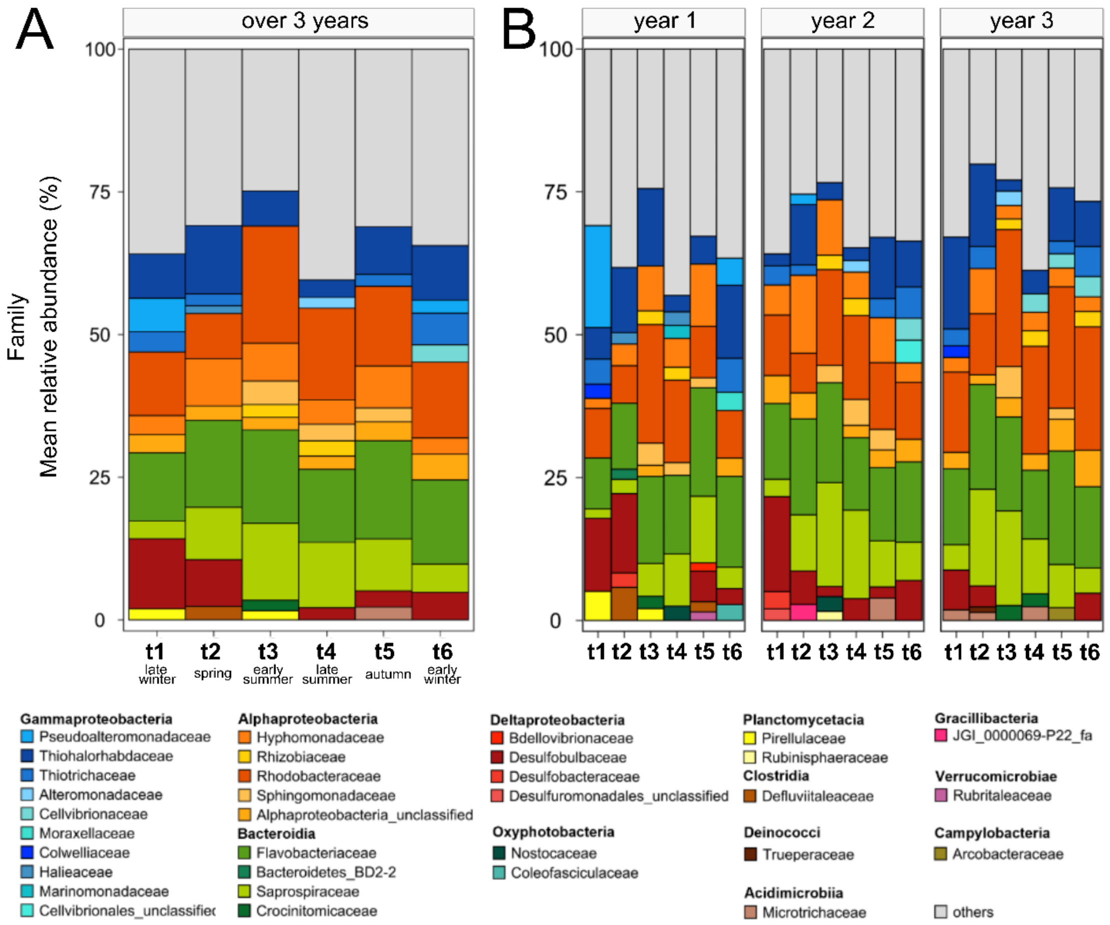

Figure S3. Microbial community composition of the most abundant families associated with the surface of the red seaweed *Gracilaria vermiculophylla*, by season (A) and by season and year (B). Sizes of the stacked bars are relative percentages, averaged over the replicates within the same group.

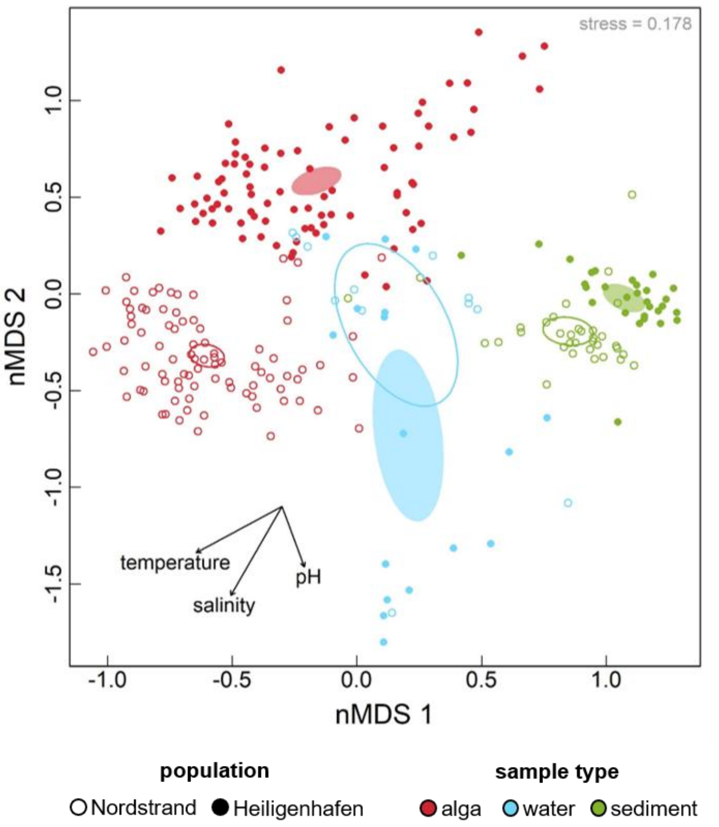

Figure S4. **nMDS with algal, water, and sediment samples.** Microbial community composition associated with the red seaweed *Gracilaria vermiculophylla* among three different sample types. The sample types alga (red), water (blue), and sediment (green) are pooled over the three collection years. The Nordstrand (North Sea) population is visualized with empty and Heiligenhafen (Baltic Sea) with filled dots and ellipses, respectively. The ellipses are represented with a 95% confidence interval. Additionally, the abiotic factors temperature, salinity, and pH are plotted. The stress value is given in the upper right corner. The microbial diversity is represented by the rarefied OTU read counts.

Table S1. PERMANOVA table for the microbial composition within all substrates (alga, water, sediment).

	df	*Sums Sqs.*	*Mean Sqs.*	*F-value*	R^2	*p-value*[1]
LSD	1	10.918	10.918	57.982	0.107	**< 0.001**
season	5	4.689	0.938	4.980	0.046	**< 0.001**
year	2	1.548	0.774	4.111	0.015	**< 0.001**
population	1	5.416	5.416	28.762	0.053	**< 0.001**
substrate	2	11.909	5.955	31.622	0.117	**< 0.001**
season:year	10	4.507	0.451	2.394	0.044	**< 0.001**
season:population	5	3.410	0.682	3.621	0.033	**< 0.001**
year:population	2	1.250	0.625	3.320	0.012	**< 0.001**
season:substrate	9	4.717	0.524	2.783	0.046	**< 0.001**
year:substrate	4	2.280	0.570	3.027	0.022	**< 0.001**
population:substrate	2	3.893	1.947	10.337	0.038	**< 0.001**
season:year:population	10	3.839	0.384	2.039	0.038	**< 0.001**
season:year:substrate	14	4.473	0.320	1.697	0.044	**< 0.001**
season:population:substrate	8	3.001	0.375	1.992	0.029	**< 0.001**
year:population:substrate	4	1.545	0.386	2.051	0.015	**< 0.001**
season:year:population:substrate	7	1.788	0.255	1.357	0.018	0.002
Residuals	175	32.953	0.188		0.323	
Total	261	102.136			1	

[1]*Terms were sequentially tested (from first to last) and p-values were obtained with 9999 permutations*
Abbreviations: Permutational Analysis of Variance (PERMANOVA), Log-transformed sequencing depth (LSD)

Table S2. PERMANOVA table for the microbial composition solely within the substrate alga is shown.

	df	*Sums Sqs.*	*Mean Sqs.*	*F-value*	R^2	*p-value*[1]
LSD	1	7.206	7.206	37.777	0.118	**< 0.001**
season	5	5.596	1.119	5.867	0.092	**< 0.001**
year	2	1.676	0.838	4.393	0.028	**< 0.001**
population	1	6.659	6.659	34.907	0.109	**< 0.001**
season:year	10	4.653	0.465	2.439	0.076	**< 0.001**
season:population	5	3.964	0.793	4.156	0.065	**< 0.001**
year:population	2	1.515	0.758	3.972	0.025	**< 0.001**
season:year:population	10	4.260	0.426	2.233	0.070	**< 0.001**
Residuals	133	25.371	0.191		0.417	
Total	169	60.900			1	

[1]*Terms were sequentially tested (from first to last) and p-values were obtained with 9999 permutations*
Abbreviations: Permutational Analysis of Variance (PERMANOVA), Log-transformed sequencing depth (LSD)

Table S3. PERMANOVA table for the predicted functional composition solely within the substrate alga is shown.

	df	*Sums Sqs.*	*Mean Sqs.*	*F-value*	R^2	*p-value*[1]
LSD	1	17.036	17.036	237.475	0.544	**< 0.001**
season	5	1.136	0.227	3.167	0.036	**0.001**
year	2	0.499	0.250	3.478	0.016	**0.008**
population	1	0.214	0.214	2.979	0.007	**0.041**
season:year	10	1.142	0.114	1.591	0.036	**0.043**
season:population	5	0.438	0.088	1.220	0.014	0.254
year:population	2	0.110	0.055	0.770	0.004	0.553
season:year:population	10	1.223	0.122	1.705	0.039	**0.024**
Residuals	133	9.541	0.072		0.304	
Total	169	31.338			1	

[1]Terms were sequentially tested (from first to last) and p-values were obtained with 9999 permutations
Abbreviations: Permutational Analysis of Variance (PERMANOVA), Log-transformed sequencing depth (LSD)

Table S4A. ANOVA table for richness based on OTUs and KOs.

	df	*OTUs*		*KOs*	
		LR Chisq.	*p-value*	*LR Chisq.*	*p-value*
LSD	1	253.434	**< 0.001**	545.53	**< 0.001**
season	5	37.331	**< 0.001**	112.80	**< 0.001**
year	2	18.740	**< 0.001**	61.03	**< 0.001**
population	1	18.565	**< 0.001**	7.12	**0.008**
season:year	10	35.137	**< 0.001**	17.32	0.068
season:population	5	22.422	**< 0.001**	7.18	0.208
year:population	2	0.980	0.613	5.44	0.066
season:year:population	10	14.703	0.143	34.18	**< 0.001**

Abbreviations: Log-transformed sequencing depth (LSD)

Supplementary Table S4B. Post-hoc pair-wise comparisons within the factor season (t1 – t6) for richness based on OTUs and KOs.

Contrast	*df*	*OTUs*		*KOs*	
		t-value	*p-value*	*t-value*	*p-value*
t1:t2	133	2.469	0.1408	2.622	0.0393
t1:t3	133	4.847	**0.0001**	8.834	**< 0.0001**
t1:t4	133	-0.055	1.0000	4.060	**0.0012**
t1:t5	133	2.057	0.3162	3.390	**0.0116**
t1:t6	133	1.441	0.7022	-0.142	1.0000
t2:t3	133	2.091	0.2982	5.782	**< 0.0001**
t2:t4	133	-2.616	0.1006	1.270	0.8007
t2:t5	133	-0.591	0.9915	0.544	0.9942
t2:t6	133	-1.195	0.8384	-2.886	0.0508
t3:t4	133	-5.085	**< 0.0001**	-4.832	**0.0001**
t3:t5	133	-3.582	**0.0461**	-5.760	**< 0.0001**
t3:t6	133	2.210	**0.0062**	-9.447	**< 0.0001**
t4:t5	133	1.556	0.2401	-0.796	0.9678
t4:t6	133	-0.659	0.6286	-4.481	**0.0002**
t5:t6	133	0.580	0.9860	-3.737	**0.0037**

Table S5A. ANOVA table for evenness (Probability of Interspecific Encounter) based on OTUs and KOs.

	Df	*OTUs*		*KOs*	
		LR Chisq.	***p-value***	***LR Chisq.***	***p-value***
season	5	86.574	**< 0.001**	39.519	**< 0.001**
year	2	8.234	**0.01629**	7.628	**0.02206**
population	1	63.915	**< 0.001**	19.463	**< 0.001**
season:year	10	36.697	**< 0.001**	22.587	**0.01238**
season:population	5	30.198	**< 0.001**	25.808	**< 0.001**
year:population	2	0.964	0.61755	2.265	0.32221
season:year:population	10	40.915	**< 0.001**	40.085	**< 0.001**

Supplementary Table S5B. Post-hoc pair-wise comparisons within the factor season (t1 – t6) for for evenness (Probability of Interspecific Encounter) based on OTUs and KOs.

Contrast	***df***	***OTUs***		***KOs***	
		t-value	***p-value***	***t-value***	***p-value***
t1:t2	133	-2.214	0.2385	2.865	0.0536
t1:t3	133	-2.327	0.1906	3.062	**0.0312**
t1:t4	133	-8.109	**< 0.001**	-0.764	0.9729
t1:t5	133	-4.622	**< 0.001**	2.073	0.3078
t1:t6	133	-1.466	0.6864	-1.126	0.8697
t2:t3	133	0.073	1.0000	-0.041	1.0000
t2:t4	133	-5.672	**< 0.001**	-3.659	**0.0048**
t2:t5	133	-2.169	0.2593	-0.982	0.9229
t2:t6	133	0.903	0.9452	-4.028	**0.0013**
t3:t4	133	-6.235	**< 0.001**	-3.936	**0.0018**
t3:t5	133	-2.442	0.1495	-1.026	0.9086
t3:t6	133	0.904	0.9449	-4.345	**< 0.001**
t4:t5	133	3.836	**0.0026**	2.921	**0.0461**
t4:t6	133	7.131	**< 0.001**	-0.364	0.9991
t5:t6	133	3.347	0.0133	-3.319	0.0145

Table S6. Epiphytic cores (see separate Excel file)
Table S7. Higher taxonomic rank cores (see separate Excel file)
